# Design and Simulation for Minimizing Non-Radiative Recombination Losses in CsGeI_2_Br Perovskite Solar Cells

**DOI:** 10.3390/nano14201650

**Published:** 2024-10-14

**Authors:** Tingxue Zhou, Xin Huang, Diao Zhang, Wei Liu, Xing’ao Li

**Affiliations:** 1 Institute of Advanced Materials, Jiangsu Provincial Engineering Research Center of Low Dimensional Physics and New Energy, School of Science, Nanjing University of Posts and Telecommunications (NJUPT), Nanjing 210023, China; zhoutingxue@outlook.com (T.Z.); 19155519686@163.com (X.H.);; 2 School of Physics and Electronic Information, Jiangsu Second Normal University, Nanjing 210023, China

**Keywords:** CsGeI_2_Br, perovskite solar cells, defects, non-radiative recombination, numerical simulation

## Abstract

CsGeI_2_Br-based perovskites, with their favorable band gap and high absorption coefficient, are promising candidates for the development of efficient lead-free perovskite solar cells (PSCs). However, bulk and interfacial carrier non-radiative recombination losses hinder the further improvement of power conversion efficiency and stability in PSCs. To overcome this challenge, the photovoltaic potential of the device is unlocked by optimizing the optical and electronic parameters through rigorous numerical simulation, which include tuning perovskite thickness, bulk defect density, and series and shunt resistance. Additionally, to make the simulation data as realistic as possible, recombination processes, such as Auger recombination, must be considered. In this simulation, when the Auger capture coefficient is increased to 10^−29^ cm^6^ s^−1^, the efficiency drops from 31.62% (without taking Auger recombination into account) to 29.10%. Since Auger recombination is unavoidable in experiments, carrier losses due to Auger recombination should be included in the analysis of the efficiency limit to avoid significantly overestimating the simulated device performance. Therefore, this paper provides valuable insights for designing realistic and efficient lead-free PSCs.

## 1. Introduction

Hybrid organic–inorganic perovskite solar cells, as an efficient and low-cost photovoltaic technology for capturing solar energy, have emerged as one of the most active research fields in photovoltaic technology [1,2,3,4]. The power conversion efficiency (PCE) of single-junction PSCs has improved significantly over the past decade, increasing from the initial 3.8% to the latest certified value of 26.1% [5]. This advancement fully demonstrates the vast potential and rapid development of PSC technology. Currently, the highest PCE achieved by this technology has matched the efficiency of some commercial solar cells on the market, such as copper indium gallium selenide (CIGS) solar cells and crystalline silicon. In the four-terminal configuration of all-perovskite stacked PSCs, the PCE has reached an impressive level above 27% [6], and perovskite-silicon stacked solar cells have even surpassed the 34% efficiency threshold [7]. The notable advancements are attributed to the superior characteristics of metal halide perovskite, as well as the rapid assimilation of lessons from other photovoltaic technologies, such as dye-sensitized solar cells, organic photovoltaics, and silicon solar cells. However, despite the remarkable breakthrough in performance, the stability issue of PSC technology and lead toxicity still limits its commercialization. To overcome this challenge, researchers have begun to explore new material systems, among which lead-free cesium compounds have attracted much attention for their excellent photovoltaic performance and tunable energy-band structure. Cesium lead-free halide PSCs not only have higher photovoltaic conversion efficiency but also have significant advantages in terms of cost and material safety, which are expected to promote the sustainable development of the photovoltaic industry [8,9].

CsGeI_2_Br has attracted considerable interest within the perovskite industry due to its non-toxic composition, together with its 1.579 eV band gap, high absorption coefficient (over 10^5^ cm^−1^) in the visible range, and conductivity [10,11]. Recent studies have shown that compared to organic–inorganic hybrid perovskites, cesium lead-free halide perovskites exhibit higher thermal stability. Among all lead-free inorganic PSCs, CsSnI_3_ achieves the highest PCE, reaching 11.21% [12]. Without considering non-radiative recombination, the simulation by Joy Sarkar and others using the solar cell capacitance simulator-one dimension (SCAPS-1D) version 3.3.10 employed Spiro-OMeTAD as the hole transport layer (HTL), TiO_2_ as the electron transport layer (ETL), and CsGeI_2_Br as the perovskite layer, achieving a device efficiency of 25.15%, open-circuit voltage (V_oc_) of 1.23 V, short-circuit current density (J_sc_) of 26.42 mA/cm^2^, and fill factor (FF) of 77.74% [11]. Nonetheless, it is inescapable that non-radiative recombination losses are present across all categories of solar cells, mainly due to phenomena such as tail recombination, electron–phonon coupling, Auger recombination, and recombination facilitated by defects [13]. The performance characteristics of the record device, including the V_oc_, J_sc_, FF, and PCE, are caused to deviate from the radiation limit by these losses [14,15,16,17]. Additionally, non-radiative recombination centers usually accelerate the degradation of perovskite absorbers, which hinders their development [18,19]. In order to further improve the performance of the device within the Shockley–Queisser (SQ) radiation limit and to improve the long-term stability of solar cells, it is extremely important to reduce all types of non-radiative recombination losses.

To unlock the full potential of CsGeI_2_Br-based PSCs, we have investigated their working mechanisms and optimization outcomes utilizing numerical simulations. The performance of PSCs is comprehensively assessed through a detailed examination of the optical/electrical parameters, which include perovskite thickness, bulk defect density, series and shunt resistance, and Auger recombination. The simulation results suggest that (1) bulk defects are identified as important factors limiting the device performance and (2) high shunt resistance, low series resistance, and appropriate Auger recombination coefficients reduce non-radiation recombination losses and improve a device’s V_oc_ and FF, making it approach the theoretical calculated SQ limit efficiency. Therefore, this study provides guidance for researchers to optimize PSCs and ultimately approach the photovoltaic impact of the SQ radiation limit.

## 2. Theoretical Methods

SCAPS-1D is a widely used simulation software designed specifically for studying the electrical and optical properties of thin-film solar cells [20]. Detailed analyses of critical solar cell processes, such as carrier transport and recombination, are supported in one-dimensional structures. In this study, SCAPS-1D simulation software (Version 3.3.10) was employed. Given the increasing importance of numerical simulation for understanding, designing, and optimizing high-efficiency solar cells, the software was utilized to intuitively analyze cell parameters, enabling rapid and accurate predictions of cell performance. This, in turn, guided experiments and optimized the research and development process [21]. SCAPS-1D simulations are primarily based on Poisson’s equation, the continuity equations for electrons and holes (Equations (1)–(3)), constitutive equations (Equations (10) and (11)), and boundary conditions for semiconductor devices [22].

(1) $$\frac{\partial }{\partial x}\left(\varepsilon_{0}\varepsilon \frac{\partial \psi (x)}{\partial x}\right)=-q\left(p-n+N_{D}^{+}-N_{A}^{-}+p_{t}-n_{t}\right)$$

(2) $$-\frac{\partial J_{n}}{\partial x}-U_{n}+G=\frac{\partial n}{\partial t}$$

(3) $$-\frac{\partial J_{p}}{\partial x}-U_{p}+G=\frac{\partial p}{\partial t}$$

where *q* is the amount of element charge; *ε* and *ε*_0_ stand for relative permittivity and free space permittivity, respectively; n and p are the electron and hole concentrations; *Ψ* is the potential; *p_t_* and *n_t_* are the hole and electron density distributions; *N_D_^+^* and *N_A_* are the donor and acceptor concentrations; *J_n_* and *J_p_* are the electron and hole current density; *G* is the carrier generation rate; and *U_n_* and *U_p_* are the electron and hole recombination rate.

In general, the boundary conditions of hole and electron flow density and device potential are determined, the concentration of electron and hole and electric field can be solved, and other relevant working parameters of cell devices can be further obtained. Set the potential at the back contact to 0, then the boundary conditions at the back contact are as follows:

(4) φ(L)=0

(5) $$J_{n}(L)=-qS_{n^{\prime }}^{b}[n^{\prime }(L)-n_{eq}(L)]$$

(6) $$J_{p}(L)=qS_{p^{\prime }}^{b}[p^{\prime }(L)-p_{eq}(L)]$$

The boundary conditions at the front contact are as follows:

(7) $$\varphi (0)=\varphi_{f}-\varphi_{b}+V_{app}$$

(8) $$J_{n}(0)=qS_{n^{\prime }}^{f}[n^{\prime }(0)-n_{eq}(0)]$$

(9) $$J_{p}(0)=-qS_{p^{\prime }}^{f}[p^{\prime }(0)-p_{eq}(0)]$$

The superscripts *f* and *b* denote the front and back electrodes, respectively, while *V_app_* represents the applied bias across the device. *ψ_f_* and *ψ_b_* indicate the work functions of the front and back contacts. *n_eq_*(*L*), *p_eq_*(*L*), *n_eq_*(0), and *p_eq_*(0) refer to the equilibrium concentrations of free electrons and holes at *x = L* and *x =* 0, respectively. *p*’ and *n’* are the carrier concentrations at the contacts, and the surface recombination rates of holes and electrons at the front and back electrodes are denoted as *S_p′_^f^*, *S_n′_^f^*, *S_p′_^b^*, and *S_n′_^b^*, respectively.

The constitutive equations are as follows:

(10) $$J_{n}=-\frac{\mu_{n}n}{q}\frac{\partial E_{Fn}}{\partial x}$$

(11) $$J_{p}=+\frac{\mu_{p}p}{q}\frac{\partial E_{Fp}}{\partial x}$$

The electron and hole quasi-Fermi levels are represented by *E_Fn_* and *E_Fp_*; *μ_n_* and *μ_p_* are electron and hole mobility. Together with appropriate boundary conditions at the interfaces and contacts, this results in a system of coupled differential equations in (*Ψ*, *n*, *p*) (Equations (1)–(3)) or (*Ψ*, *E_Fn_*, *E_Fp_*) (Equations (10) and (11)).

**Figure 1:**
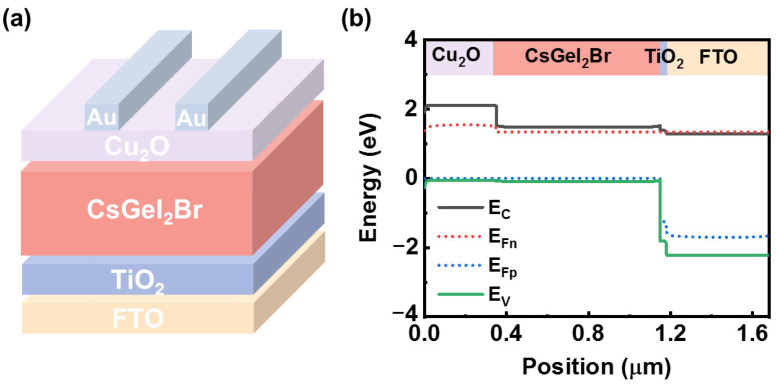
(**a**) Schematic diagram of the perovskite device used for this simulation. (**b**) Energy−band alignment of ETL and HTL with CsGeI_2_Br perovskite and FTO.

The simulation uses an AM1.5G light source of 1000 W/cm^2^ for constant lighting, and the temperature is set to 300 K. The device structure type used in the simulation is n-i-p, typically consisting of an FTO substrate, an ETL, a perovskite layer, an HTL, as well as a back-contact electrode system, and it is illustrated in Figure 1a, where Cu_2_O is chosen as the HTL and TiO_2_ as the ETL. Figure 1b shows the energy-level arrangement of the designed CsGeI_2_Br with the charge-transport layer. While series resistance (*R_s_*) is 5 Ω cm^2^, shunt resistance (*R_sh_*) is 5000 Ω cm^2^. The parameters for CsGeI_2_Br, Cu_2_O, and TiO_2_ are displayed in Table 1. Additionally, Table 2 presents the parameters for the interface defects between the CsGeI_2_Br layer and the transport layers, and these parameters are based on previous experiments and the published literature.

## 3. Results and Discussion

### 3.1. Dependence of Perovskite Thickness

**Figure 2:**
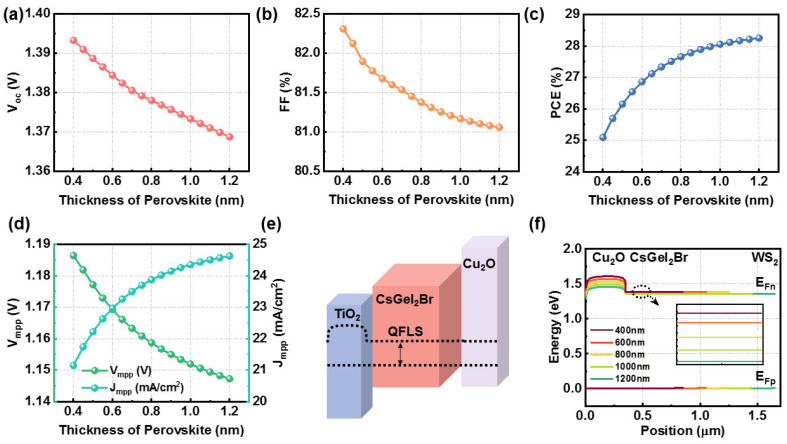
Effects of the various perovskite thicknesses on device performance: (**a**) *V_oc_*, (**b**) FF, (**c**) PCE, (**d**) *V_mpp_* and *J_mpp_*. (**e**) Simulation of QFLS and *V_oc_* of PSCs. (**f**) Energy−band diagram of the Fermi energy levels of electrons and holes that change with various perovskite thicknesses.

The adjustment of perovskite material thickness significantly impacts its electrical, optical, and stability properties. Being too thin or too thick can reduce carrier transport efficiency, cause energy-level mismatches, degrade interface charge-transfer efficiency, and compromise stability. Precise thickness control is crucial for optimizing the performance of PSCs, aiming for the optimal PCE and stability [27,28]. This study examined thickness variations between 400 and 1200 nm, as depicted in Figure 2, which suggest the following: (i) Figure 2a reveals that as thickness increases, the *V_oc_* decreases from 1.37 V to 1.40 V due to an enhancement in the spectral response in the long-wavelength region [29]. (ii) With increasing thickness, both the *J_sc_* and PCE show an upward trend, and the PCE increases from 25.09% to 28.25%. (iii) Conversely, the *FF* decreases as the thickness increases.

In detail, this is an important indicator of the conversion efficiency of a solar cell or electronic device. There is a specific mathematical relationship between the fill factor and parameters such as the *V_oc_*, *J_sc_*, *V_mmp_*, and *J_mmp_*, and usually the *FF* is calculated as follows:

(12) $$FF=\frac{V_{mpp}\times J_{mpp}}{V_{oc}\times J_{sc}}$$

where *V_mpp_* is maximum power voltage and *J_mpp_* is maximum power current. Specifically, as the perovskite thickness increases, there is an increase in light scattering and absorption within the device, leading to a decrease in the *V_oc_* and *J_sc_*. Additionally, with the increased thickness, the carrier transport distance within the device also increases, resulting in a reduction in *V_mmp_* and an increase in *J_mmp_*. These changes affect the calculated *FF*; thus, the overall trend is a decrease in the *FF* with increasing thickness.

To further comprehend the reason for the variation in the *V_oc_*, quasi-Fermi level splitting (QFLS) is a key factor impacting the *V_oc_* [30], with Equation (9) illustrating its relationship with the Voc. QFLS is defined as the difference between the electron quasi-Fermi level (*E_Fe_*) and the hole quasi-Fermi level (*E_Fh_*) (Figure 2e) [30].

(13) $$V_{oc}=\frac{QFLS}{q}=\frac{1}{q}(E_{Fe}-E_{Fh})$$

Figure 2f shows that as thickness increases, *E_Fn_* remains constant while *E_Fp_* decreases, leading to reduced QFLS and *V_oc_*. Thus, perovskite layer thickness significantly affects both QFLS and *V_oc_*. While greater thickness improves light absorption, it also increases non-radiative recombination and series resistance, further reducing QFLS and *V_oc_*. Studies suggest the optimal thickness is below 1000 nm. Based on experimental validation, 900 nm offers an ideal balance, enhancing absorption efficiency while minimizing recombination losses, ensuring both efficiency and stability [31,32].

### 3.2. Dependence of Perovskite Bulk Defect Density

**Figure 3:**
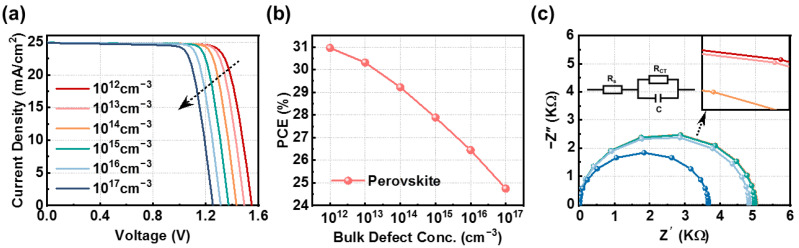
(**a**) Plots of the current–voltage (J−V) curves with different N_t_ and (**b**) the corresponding EQE curves. (**c**) The impedance of optimized PSCs with different N_t_ (In the magnified image, magenta represents 10^12^ cm^−3^, pink represents 10^13^ cm^−3^, and orange represents 10^14^ cm^−3^).

One of the primary impediments to the enhancement of PSC performance is the non-radiative recombination loss of bulk carriers. To enhance the device’s performance and stability, it is crucial to efficiently minimize the surface-related non-radiative recombination losses [33,34]. The bulk defect density (N_t_) in CsGeI_2_Br-based PSCs is adjusted from 10^12^ cm^−3^ to 10^17^ cm^−3^ (Figure 3). As shown in Figure 3a, with the increase in N_t_, both the *V_oc_* and *J_sc_* decrease; however, the reduction in the *J_sc_* is relatively small. This effect is due to the increase in the concentration of defects, which accelerates the recombination process of charge carriers inside the device, consequently decreasing the count of functional carriers and, consequently, a decrease in both the *V_oc_* and *J_sc_*.

To further verify that lower N_t_ reduces interfacial charge recombination, electrochemical impedance spectroscopy (EIS) is used for measurements [35,36,37]. There are two processes that can contribute to EIS.

(14) $$Z=Z^{\prime }-jZ^{\prime \prime }$$

The impedance is a complex number *Z* that can be expressed as two parts of the real part Z′ and the imaginary part *Z*″.

(15) $$Z^{\prime }=R_{S}+\frac{R_{CT}}{1+\omega^{2}C^{2}R_{CT}^{2}}$$

Here, *R_s_* is the series resistance; *R_CT_* is the charge-transfer resistance, corresponding to the diameter of the semicircle in the Nyquist plot; and *C* is the capacitance. *ω* is the frequency.

Figure 3c shows the Nyquist curve for the CsGeI_2_Br device. In the context of EIS, key parameters such as the *R_s_*, the *R_CT_*, and the recombination resistance (R_rec_) can be derived from the measurement data. *R_s_* is typically calculated from the initial intercept on the Z′ axis of the high-frequency arc in a Nyquist plot. At high frequencies, R_tr_ and the low-frequency R_rec_ correspond to the charge transfer at the HTL/perovskite interface and the recombination at the perovskite/ETL interface, respectively. Under light and high photoconductivity conditions, the R_rec_ predominantly contributes, given the negligible contribution of the *R_CT_* to the impedance response in high-performance PSCs [10,38]. The dominant semicircle in the Nyquist plot shown in Figure 3c is associated with the R_rec_. As the N_t_ increases, the R_rec_ values corresponding to CsGeI_2_Br devices gradually decrease. The smaller the R_rec_, the easier it is for carrier recombination within the perovskite, resulting in fewer electrons and holes being collected by the carrier layer, thereby reducing device performance [39]. Considering low doping is challenging, we chose N_t_ = 10^14^ cm^−3^ as the optimal value for enhanced device performance.

### 3.3. Dependence of Perovskite Series and Shunt Resistance

In PSCs, *R_s_* and *R_sh_* lead to energy loss, thereby diminishing overall efficiency. This encompasses the resistances contributed by the back contacts (Au), HTL, perovskite layer, ETL, and front contacts (FTO). These resistance effects are challenging to eliminate completely within solar cells. Therefore, it is important to conduct a thorough examination of these factors in order to enhance the efficiency of PSCs [40,41].

**Figure 4:**
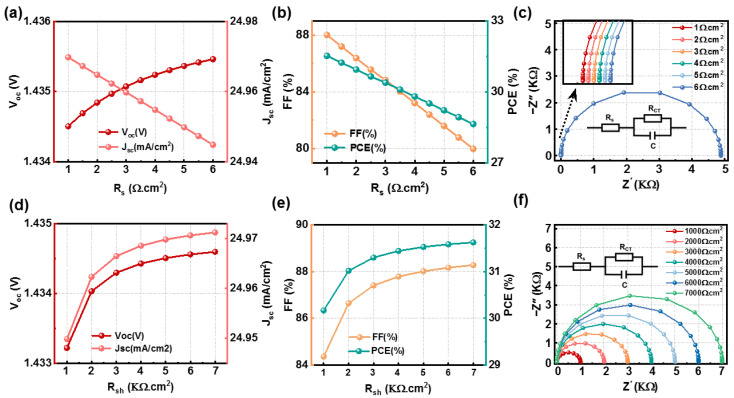
(**a**,**b**) Effect of the variation in R_s_ on device performance and (**c**) the corresponding impedance. (**d**–**f**) Effect of the variation in R_sh_ on device performance and (**c**) the corresponding impedance.

In this simulation study, *R_s_* was varied from 1 to 6 Ω cm^2^ and *R_sh_* from 1000 to 7000 Ω cm^2^, with the changes in photovoltaic parameters being examined, as depicted in Figure 4a,b from which we can conclude the following: (i) When the series resistance *R_s_* increases, the *V_oc_* of the device only experiences a slight change, going from 1.4345 V to 1.4355 V, while there is a slight increase noted in the *J_sc_*, moving from 24.95 mA/cm^2^ to 24.97 mA/cm^2^. Conversely, the reductions in the FF and PCE are notably more pronounced, decreasing from 80.00% to 88.01% and from 28.64% to 31.53%, respectively. As illustrated in Figure 4c, the increase in *R_s_* results in a reduction in the semicircle radius in the Nyquist plot, indicating a weakening of the electric field strength. This leads to an increased likelihood of electron and hole recombination before reaching the electrodes due to interactions with defects, impurities, or interfaces within the device, thereby diminishing the overall efficiency of the PSC, so we chose *R_s_* = 1 Ω cm^2^ as the optimal value for enhanced device performance. (ii) With the increase in *R_sh_* (*R_s_* = 1 Ω cm^2^), the *V_oc_* and *J_sc_* are slightly affected, with the *V_oc_* dropping from 1.433V to 1.435V and the *J_sc_* rising from 24.95 mA/cm^2^ to 24.97 mA/cm^2^. In contrast, *R_sh_* increases cause more significant changes in the FF and PCE, with the FF decreasing from 84.37% to 88.28% and the PCE from 30.17% to 31.62%. Figure 4f illustrates that the Nyquist plot’s semicircle expands with rising *R_sh_*, reflecting an increasing R_rec_ for the CsGeI_2_Br device. A higher R_rec_ reduces recombination in the perovskite, leading to more collected electrons and holes in the charge-transport layer, improving device performance, so the optimal research parameter determined in this simulation is *R_sh_* = 7000 Ω cm^2^. Therefore, under conditions of high *R_sh_* and low *R_s_*, the device performance of the CsGeI_2_Br-based PSCs is better. Under this condition, the *V_oc_* = 1.44V, *J_sc_* = 24.97 mA/cm^2^, FF = 88.28%, and PCE = 31.62%.

### 3.4. Dependence of Perovskite Auger Recombination

Auger recombination represents a crucial limiting factor in the efficiency of PSCs, precipitating a range of detrimental effects, including carrier recombination losses, interface energy-level mismatches, the augmentation of defect states, and limitations on charge transport. The final section will provide a comprehensive investigation of the impact of Auger recombination on device performance [42,43]. For Auger recombination process, U_Auger_ is expressed as follows:

(16) $$U_{Auger}=(c_{n}^{A}n+c_{p}^{A}p)(np-n_{i}^{2})$$

The parameters *C_n_^A^* and *C_p_^A^* represent the Auger recombination coefficients for electrons and holes, respectively. Here, *n* and *p* denote the densities of electrons and holes, while *n_i_* is the intrinsic carrier density.

**Figure 5:**
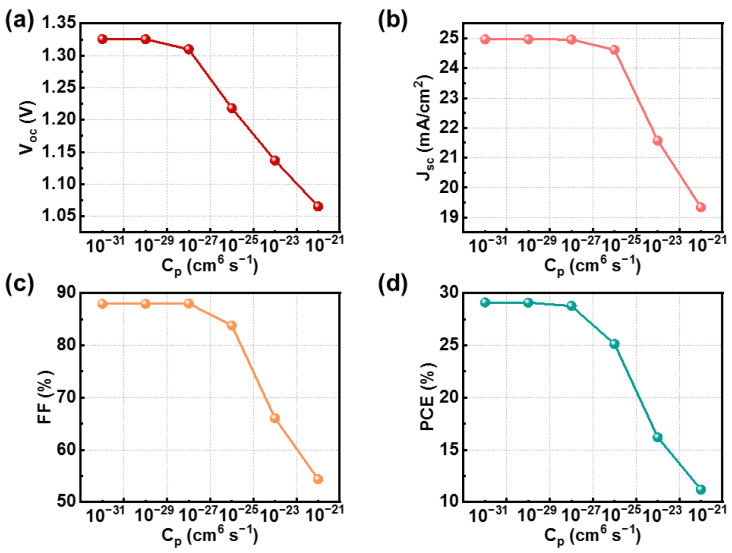
(**a**–**d**) Effect of the variation Auger recombination coefficient on device performance.

When analyzing the device characteristics using the SCAPS-1D program, the radiative recombination coefficient (3 × 10^−11^ cm^3^ s^−1^) and Auger electron/hole capture coefficient are taken into account; we specialize in the effect of Auger recombination on the performance of PSCs. In our research, the radiative recombination coefficient is found to be less than 10^−20^ cm^6^ s^−1^. The variation in PSC photovoltaic parameters with the changes in Auger electron/hole capture coefficients (C_n_/C_p_) is depicted in Figure 5. This study reveals that the photovoltaic performance parameters remained essentially unchanged within the range of 10^−31^ to 10^−29^ cm^6^ s^−1^, with the PCE dropping from 29.11% to 29.10% However, as the Auger recombination coefficient exceeded 10^−27^ cm^6^ s^−1^, these parameters began to decline, with the PCE dropping from 28.78% to 11.20%. This decline is attributed to the increased Auger recombination and carrier recombination, which in turn limited the carrier transport properties. The reduction in carrier transport properties can lead to an increase in the device’s R_s_, consequently diminishing cell efficiency. Hence, it is advised to maintain the C_p_ of PSCs at a level less than 10^−29^ cm^6^ s^−1^ to reduce the effect of Auger recombination on photovoltaic efficiency. This value aligns with the findings of Ali K. Al-Mouso et al., indicating its feasibility [43].

## 4. Conclusions

In this study, we elucidated the working mechanisms and optimization outcomes of CsGeI_2_Br-based PSCs through rigorous numerical simulation. A detailed explanation was given on how parameters such as perovskite thickness, bulk defect density, series and shunt resistance, and Auger recombination affect device performance by using a TiO_2_/CsGeI_2_Br/Cu_2_O/Au device structure and a series of studies. Based on the simulation results, a low bulk defect density, high R_sh_, low R_s_, and appropriate Auger recombination coefficient are crucial for the high efficiency of CsGeI_2_Br-based PSCs. Additionally, when the Auger capture coefficient is increased to 10^−29^ cm^6^ s^−1^, the efficiency drops sharply from 31.62% (without considering Auger recombination) to 29.10%. The results suggest that the carrier lifetime in the PSCs should be reduced to below 10^−29^ cm^6^ s^−1^ to minimize the Auger recombination. Only by suppressing interfacial defect recombination can the full potential of the perovskite absorber be achieved. The ultimate goal is to push this technology toward its radiative limit by suppressing defects in the perovskite bulk or at grain boundaries.

## Figures and Tables

**Table 1:** Material parameters of HTL, ETL, and perovskite required in the device modeling.

Material Parameters	FTO [23]	TiO_2_ [24]	Cu_2_O [25,26]	CsGeI_2_Br [11]
Thickness (nm)	500	30	350	800
Band gap, *E_g_* (eV)	3.5	3.2	2.17	1.579
Electron affinity (eV)	4.0	3.9	3.2	3.76
Dielectric permittivity (relative)	9.0	9.0	7.1	18.0
CB effective density of states (1/cm^3^)	2.2 × 10^18^	2 × 10^18^	2.02 × 10^17^	9.65 × 10^17^
VB effective density of states (1/cm^3^)	1.8 × 10^19^	1.8 × 10^19^	1.1 × 10^19^	1.04 × 10^18^
Electron thermal velocity (cm/s)	1 × 10^7^	1 × 10^7^	1 × 10^7^	1 × 10^7^
Hole thermal velocity (cm/s)	1 × 10^7^	1 × 10^7^	1 × 10^7^	1 × 10^7^
Electron mobility (cm^3^/Vs))	20	20	200	20
Hole mobility (cm^3^/Vs)	10	10	80	20
Shallow uniform donor density N_D_ (1/cm^3^)	1.8 × 10^19^	9 × 10^16^	0	0
Shallow uniform acceptor density N_A_ (1/cm^3^) Total density (1/cm^3^)	0	0	1 × 10^18^	2 × 10^16^
	1 × 10^15^	1 × 10^15^	1 × 10^15^	1 × 10^15^

**Table 2:** Material parameters of interface defect layers.

Interface	Defect Type	Capture Cross Section: Electrons/Holes (cm^2^)	Energetic Distribution	Reference for Defect Energy Level	Total Density (cm^−3^)
ETL/CsGeI_2_Br	neutral	1 × 10^−19^/1 × 10^−19^	single	above the VB maximum	1 × 10^10^
CsGeI_2_Br/HTL	neutral	1 × 10^−19^/1 × 10^−19^	single	above the VB maximum	1 × 10^10^

## Data Availability

The original contributions presented in the study are included in the article, further inquiries can be directed to the corresponding authors.
